# Lessons from the COVID-19 pandemic for future primary care: a qualitative interview study with GPs and medical practice assistants in Germany

**DOI:** 10.3399/BJGPO.2025.0075

**Published:** 2026-06-17

**Authors:** Kahina J Toutaoui, Marius T Dierks, Christoph Heintze, Lisa Kümpel, Doreen Kuschick, Liliana Rost, Florian Wolf, Susanne Doepfmer

**Affiliations:** 1 Institute of General Practice and Family Medicine, Charité - Universitätsmedizin Berlin, Corporate Member of Freie Universität Berlin and Humboldt-Universität zu Berlin, Berlin, Germany; 2 Institute of General Practice and Family Medicine, Jena University Hospital, Friedrich Schiller University, Jena, Germany

**Keywords:** COVID-19, learning, pandemics, primary health care, telemedicine, workload

## Abstract

**Background:**

The COVID-19 pandemic necessitated significant adaptations for general practices, affecting healthcare services and workload. The mixed-methods study VeCo-Praxis was designed to investigate these changes, focusing on non-COVID-related services in three German regions. This article is based on a qualitative interview study with general practitioners (GPs) and medical practice assistants (MPAs).

**Aim:**

To explore the perspectives of GPs and MPAs regarding their work and role during the pandemic and what conclusions they draw for primary care in the aftermath of the pandemic.

**Design & setting:**

A qualitative study with 15 GPs and 15 MPAs from three federal German states (August 2022–September 2023).

**Method:**

We conducted semi-structured telephone interviews and performed thematic analysis described by Braun and Clarke.

**Results:**

In this article, we focus on three key aspects: 1) the evolving role of GPs and MPAs as central healthcare coordinators; 2) frustrations stemming from inadequate collaboration with specialists, health authorities, and hospitals; and 3) conclusions and outlook for the aftermath of the pandemic. GPs reported an increased sense of responsibility but also greater exhaustion, while MPAs felt undervalued, particularly in comparison to hospital nurses. While telemedicine was helpful, both groups criticised the administrative workload, the lack of timely communication from authorities, and the lack of support with pandemic-related tasks to ensure general patient care.

**Conclusion:**

Following the pandemic it is understood that GPs and MPAs must be adequately supported in their roles, interdisciplinary collaboration must be improved, and information flows and administrative processes must be more efficient to assure better pandemic preparedness in primary care.

## How this fits in

General practices had to make significant adjustments during the COVID-19 pandemic to ensure pandemic-related and general patient care. This study focuses on the perspectives of two important professional groups (GPs and medical practice assistants [MPAs]) on their work during the pandemic and the lessons learned for future primary care in Germany. In addition to already known burdens, the heavy workload of GPs and MPAs caused by taking over tasks from other healthcare professionals and institutions is emphasised in particular. Clarifying responsibilities and creating fast information channels and easily accessible communication within the healthcare system are highlighted as key elements for better pandemic preparedness.

## Introduction

During the COVID-19 pandemic, general practices in Germany had to make significant adjustments to their organisational processes and the healthcare services they offered.^1–3^ As far as possible, the infrastructure of the practices had to be adapted to ensure enough infection control equipment in every consulting room.^2^ At the beginning of the pandemic, preventive and routine services were often delayed or postponed^3^ due to concerns about infections, upcoming changes in the healthcare system,^4^ and to provide more space for acute and urgent consultations.^3^ The pandemic also led to a shift in patient contact from face-to-face consultations to telemedicine^3^ and telephone consultations, with certain patient groups, such as the chronically ill, being particularly affected.^5^

The additional workload for staff in GP practices was substantial.^6,7^ Additional consultation hours were offered and working hours were extended.^8^ Consequently, many healthcare workers developed fatigue and symptoms of burnout.^9^

The COVID-19 pandemic shocked the entire healthcare system^10^ and impacted regular healthcare services.^2–5^ Possible lessons learned from the pandemic were discussed early on,^11^ including those for general practice.^12^ Given that procedures and outcomes may evolve throughout a pandemic, investigating changes in general practices throughout the COVID-19 pandemic, especially towards its later stages, was essential to understanding the full effect.^13^ To address the continued impact on health professionals after the initial acute phases, it is important to explore how staff reflect on their experiences and identify their needs, wishes, and demands for the future.

The mixed methods study VeCo-Praxis was conducted in the German practice-based research network RESPoNsE (Research Practice Network East) in the three federal states of Berlin, Brandenburg, and Thuringia. The study was designed to explore the views of GPs, MPAs, and patients on the provision and utilisation of healthcare services in GP practices during the COVID-19 pandemic,^14^ with a focus on non-COVID-related healthcare services. The aim was to investigate the effects of the pandemic on regular health care and to draw conclusions about needed changes for future care, involving all relevant target groups in GP practices. Aspects of the appropriateness of regular care services, the lack of regular care, and the sustainability of adjustments made in the general practices were explored. The study consisted of two anonymous, paper-based questionnaires filled out by GPs and MPAs^15,16^ at an interval of 12 months, in-depth qualitative interviews with GPs and MPAs, and anonymous paper-based questionnaires filled out by patients.^17^ A brief description of the characteristics of the German GP system and the role of MPAs can be found in Supplementary Information S1.

This article presents the findings from the qualitative sub-study based on the following research questions: how do GPs and MPAs perceive their own work and role during the pandemic? And what conclusions do they draw for primary care in the aftermath of the COVID-19 pandemic, for future primary care in general and in case of another pandemic?

## Method

The qualitative sub-study of the VeCo-Praxis study was intended to provide a deeper understanding of the adjustments made in GP practices during the COVID-19 pandemic for non-COVID-related healthcare services. One-time telephone interviews, following a semi-structured interview guide, were conducted to allow sufficient space for the perspectives and experiences of individual GPs and MPAs during the pandemic. KJT, a junior doctor in general medicine and research assistant with training in qualitative methods, conducted all interviews, supported by experienced qualitative researchers. KJT worked as a physician in training during the pandemic and was therefore familiar with many of the accounts provided by the interviewees from personal experience. Extensive and repeated discussions of the themes and codes within a multidisciplinary team ensured that personal experience did not narrow or bias the perspective.

### Interview guide

The interview guides (one for each professional group) were developed according to Kallio *et al*.^18^ They were based on a literature review and discussions with GPs and MPAs in the RESPoNsE practice-based research network (PBRN), particularly members of the RESPoNsE practice advisory board, composed of GPs and MPAs who provide feedback on and help shape research projects from a practice-based perspective, and within the multidisciplinary study team in the context of emerging results from the other study components. The interview guides were piloted with a member of the corresponding professional group. Due to the prolonged nature of the pandemic and the new insights of the first VeCo-Praxis questionnaire-based survey,^19^ two questions for GPs and one for MPAs were added during the study (see Supplementary Table S1).

### Recruitment and sampling

During the first VeCo-Praxis questionnaire-based survey in May 2022,^19^ 58 GP practices expressed interest in the GP interviews and 29 in the MPA interviews, with some overlap. Initially, the following purposive sampling strategy^20^ was used to obtain a broad spectrum of contexts and views: practices from all three federal states of the RESPoNsE network; practices within or outside the RESPoNsE PBRN; different locations (rural, urban, and different city districts); GPs’ gender ratio (MPA gender was not known in advance); and a maximum of one person from the practice advisory board. Practices selected according to the sampling strategy received study documents by post and, if necessary, a reminder. Eight GPs and eight MPAs did not return the study documents or did not report back without stating any reasons. As initial recruitment included too few patients from rural areas and Thuringia, we specifically sought to increase representation from these regions. Compensation for participation (EUR 100; approximately 85 GBP) was provided to reimburse time and effort.

### Data collection

Telephone interviews were conducted between August 2022 and September 2023. Written informed consent was obtained from all participants. The interviewees were free to choose the timing and location of the interviews. A few interviewees were already known to the interviewer before the start of the study through other professional contexts (the PBRN and/or involvement in the Association of General Practitioners). Field notes were taken during each interview, reflecting assumptions, emotional responses, and first thoughts for data analysis.

### Data processing and analysis

The interviews were recorded using a digital voice recorder and transcribed verbatim. The pseudonymised transcripts were not returned to the participants for comments or corrections. Potentially identifying statements were removed. The data were coded and analysed using MAXQDA 2022 software. Thematic analysis according to Braun and Clarke was employed to identify patterns or themes within the data.^21^ This process followed six phases: familiarisation with the data; generating initial codes; searching for themes; reviewing themes; defining and naming themes; and writing the report. Adherence to the method was ensured by consistently following the six phases and transparently documenting each phase in a comprehensible and traceable manner. The interviews were analysed separately for GPs and MPAs, although modifications to the key themes of each group were regularly checked to see whether they should also be made for the other group.

The analysis was carried out by a multidisciplinary team that included a resident in general medicine (KJT), a public health scientist (LK), and a nurse and public health scientist (DK). KJT was the main analyst, while DK and LK counter-coded and checked plausibility. Codes and themes were adapted several times following joint discussions. Initially, deductive codes were used based on the COVID-19 System Shock Framework.^10^ These were adapted and supplemented by inductive codes. The participants did not provide feedback on the findings.

## Results

Fifteen GPs and 15 MPAs from three federal states in Germany were interviewed. Participant characteristics are described in Table 1. The interviews were conducted in German with a duration between 21 and 52 minutes. Data saturation was reached when no new themes emerged.

**Table 1.** Sociodemographic characteristics of interviewees

Characteristic^a^	GPs (*n* = 15)	MPAs (*n* = 15)
**Age, years, mean (SD, range)**	53.3 (8.2, 37–69)	42.4 (9.3, 32–63)
**Gender**		
Man	6 (40.0)	0 (0.0)
Woman	9 (60.0)	15 (100)
**Federal state**		
Berlin	6 (40.0)	6 (40.0)
Brandenburg	5 (33.3)	4 (26.7)
Thuringia	4 (26.7)	5 (33.3)
**Location**		
Urban	8 (53.3)	11 (73.3)
Rural	7 (46.7)	4 (26.7)
**Part of the RESPoNsE practice-based research network**		
Yes	11 (73.3)	12 (80.0)
No	4 (26.7)	3 (20.0)
**Years of professional experience**		
1–5	3 (20.0)	2 (13.3)
6–10	4 (26.7)	2 (13.3)
11–20	4 (26.7)	8 (53.3)
>20	4 (26.7)	3 (20.0)
**Years working in current GP practice**		
<1 (GP / 1–2 (MPA)	1 (6.7)	1 (6.7)
1–5 (GP) / 3–5 (MPA)	4 (26.7)	4 (26.7)
6–10	3 (20.0)	4 (26.7)
11–20 (GP) / >10 (MPA)	3 (20.0)	6 (40.0)
>20 (GP)	4 (26.7)	—
**Type of practice**		
Single-handed	10 (66.7)	—
Group	5 (33.3)	—
**Type of employment**		
Self-employed (practice owner)	15 (100)	—
Employee	0 (0.0)	—
**Job title**		
MPA	—	8 (53.3)
Nurse	—	4 (26.7)
Receptionist	—	2 (13.3)
Dental practice assistance	—	1 (6.7)

^a^ *n* (%) unless otherwise stated. MPA = medical practice assistant. RESPoNsE = Research Practice Network East. SD = standard deviation.

We identified nine main themes (‘healthcare services’, ‘healthcare workforce’, ‘values in relation to practice work’, ‘information transfer/flow of information’, ‘health policy and governance’, ‘medical devices and technology’, ‘funding and finance’, ‘healthcare system in Germany’, and ‘prospects to the future’), with sub-themes that were partly different for GP and MPA interviews. In this article, we will focus on aspects that shed light on the work and role of GPs and MPAs during the COVID-19 pandemic, as well as conclusions for future GP care in general and in case of another pandemic. Focusing on these, we identified three key aspects within the main themes ‘healthcare workforce’ and ‘prospects for the future’:

- role of GPs and MPAs in the healthcare system;
- perspectives on other healthcare professionals and institutions;
- conclusions and outlook for the aftermath of the COVID-19 pandemic (see Table 2).

**Table 2.** Summary of the results: three key aspects with corresponding statements

Role of GPs and MPAs in the healthcare system	Perspectives on other healthcare professionals and institutions	Conclusions and outlook for the aftermath of the COVID-19 pandemic
GPs as healthcare coordinators, reaching their limits Pandemic reinforced importance and necessity of GPs and MPAs' work Working to the point of exhaustion, need to prioritise own health MPAs with a lot of responsibility MPAs feel unseen by political decision makers and society	Lack of direct contact Taking over tasks of other healthcare professionals and institutions (health authorities, specialist appointments, and hospitals) Establishing digital networks for better collaboration	Adaptation of in-practice procedures Switch to telemedicine, supported by introduction of remuneration Overload because of documentation requirements and new regulations during the pandemic Lack of information for GP practices Specialist practices should take over some pandemic-related services Tasks of health authorities during a pandemic (for example, swabs) GPs weigh up medical services and practice financing

MPA = medical practice assistant.

### Role of GPs and MPAs in the healthcare system

The interviewees spoke at length about their role during the pandemic, their personal experiences, and how their work was perceived by others.

GPs still saw themselves as healthcare coordinators who were responsible for patients in every situation but were concerned that their scope of responsibility was reaching its limits:

*‘I acknowledge that the GP is more or less responsible for everything, yes, you are the caretaker. But ultimately, when everyone else says: “Mhm, I cannot do that now”, then people just end up here. It’s sometimes very difficult now, in terms of what you can still manage.’* (GP 2)

The interviewees had different perspectives on their workload. Some had a positive outlook and felt that the pandemic had reinforced the importance and necessity of their work in the healthcare system:

*‘I realised during the pandemic that I appreciated the work I do now, the work of a GP, more than usual, because I realised how important talking medicine is.’* (GP 5)

Others described such a great sense of responsibility towards the patients and their work in the practice that they worked to the point of exhaustion and had to shift their priorities to maintain their health:

*‘Since the pandemic, I have really reached my limits in terms of resilience.* […] *I took the patients home with me in my thoughts* […] *and slept poorly. I actually got sick at times because the stress, the emotional strain, the workload had all become much heavier. And I had to adjust the organisation several times so that life was worth living again for me.’* (GP 4)

GPs felt overlooked by political decision makers and wanted to be involved in the decision-making process so they could check the feasibility of certain measures in advance:

*‘I would like to be heard before any decisions are made. Not because of my ego or vanity.* […] *Before decisions are made, the question is important: “Is this even realistic? Can we realise it?” I would have liked for us to be treated with respect.’* (GP 5)

MPAs felt that they were the first point of contact for patients and bore a lot of responsibility:

*‘You have a lot of responsibility in the practice and have to make a lot of decisions yourself.* […] *And I have also found that in some emergency situations, you act faster than the doctor.’* (MPA 9)

Simultaneously, they felt unseen by political decision makers and society, especially compared to nurses in hospitals:

*‘Nobody cared about us. That was the worst thing that really upset us as MPAs in general practices. They only talked about hospitals* […] *But we were at the front line. Where before we had 20 patients during consultation hour, we suddenly had 60.’* (MPA 11)

### Perspectives on other healthcare professionals and institutions

The interviewees addressed their relationships with other healthcare professionals and institutions during the pandemic, largely expressing frustration about their experiences. They complained about the lack of direct contact with specialist practices, hospitals, health authorities, and the Association of Statutory Health Insurance Physicians. Some described the communication as non-existent or poor, regardless of the pandemic. They wished for more direct interaction, especially from those considered colleagues:

*‘Most of the time, the information is passed through the patient.* […] [They say:] *“Yes, clarify that with your GP”, without there being any direct communication.* […] *No written information, only verbally via the patient.* […] *There is a lack of collegial appreciation in this regard.’* (GP 12)

Both professional groups described taking over the tasks of various other healthcare professionals and institutions during the pandemic, as health authorities were not available to provide information about quarantine regulations, consultations with specialists were cancelled, and hospitals were restrictive in their admissions:

*‘With the health authorities, you did not get through to anyone. So when it came to how long a quarantine should be. Or the reporting of patients* […] *or that patients should be informed* […] *if the swab test was positive. That did not work at all. We then took over that here in the practice.’* (GP 14)

*‘*[…] *a year and a half ago, it was quite noticeable that many of the patients we referred to specialists came back. And they said, “You cannot get an appointment with the specialists” or “the specialist has sent me back to you, you should do that”.’* (MPA 7)

*‘They* [patients] *come to the hospital and the emergency department with referral papers. Then I expect the patient to be admitted and not to be looked at after a small infusion or by the trainee and sent back home again. And we are left with the same problems as before.’* (GP 6)

GPs and MPAs requested better cooperation with other healthcare professionals and institutions by receiving written information, by direct (telephone) contact, or by establishing digital networks:

*‘That perhaps we should be networking a bit more generally. I think we are not very well connected. So many of us do not even use the option of sending data online. If you request something, you often get it in a letter form, like a patient file on paper. There is just a lack of digitalisation, digital networking, among doctors.’* (MPA 3)

### Conclusions and outlook for the aftermath of the COVID-19 pandemic

GPs and MPAs described various in-practice procedures that were adapted due to the pandemic and will be retained after the pandemic. These include separating patients with infectious diseases from other patients, wearing masks in case of respiratory infections, and reducing the number of patients in the practice at one time:

*‘We have also made some organisational changes: We now have a traffic light system in the practice. Patients can only enter the practice one by one. This is very helpful because it allows us to really concentrate on the patient in front of us.’* (MPA 4)

During the pandemic, it was important to switch part of patient contacts to telephone and video consultations. The official remuneration of telephone contacts, video consultations, and sick notes led to more frequent use of telemedicine:

*‘We have been offering this* [video consultations] *for* […] *a year and a half now.* […] *And yes, you could call and discuss something over the phone before* […] *But you could not bill it* […] *and did not get any money for it. That is why they offered very few of them, of course. When it was reimbursable, it was of course much more frequent.’* (MPA 7)

Considering the increased volume of enquiries from patients, contact by email, over the telephone, or via digital tools was perceived as relieving strain (compared to seeing patients exclusively in the practice):

*‘Then there is a phone call, then you hang up, then you have worked through it in peace, that was different and good.’* (MPA 3)

The interviewees focused on things that should have been done differently by others or within the healthcare system rather than reflecting on what they would now do differently. Pandemic-related tasks caused a high workload and were a main stress factor during the pandemic; for example, documentation requirements for COVID-19 vaccinations overloaded practice teams. GPs and MPAs had to deal with new regulations and unclear responsibilities and wished to focus more on patient care:

*‘It was this combination of medical services, billing, statistics for all sorts of authorities, and providing security for patients. It was just a mixture of all sorts of things that were crammed into one and it was just too much. I would have liked us to be able to simply vaccinate in the same way as we vaccinate all patients against tetanus and that would have been fine.’* (GP 15)* *

The interviewees criticised the inadequate flow of information during the pandemic, as the public was often informed of changes before practices. They would have liked to be informed at an early stage so that adjustments could be made before the public demanded them:

*‘It would have been better to inform the GP practices before they went public.* […] *Patients called here when they heard of a change to a rule, and of course we did not hear the news during the day and did not have any information from any medical association.’* (MPA 12)

Besides the lack of communication, some interviewees were frustrated that specialist practices did not support GP practices during the pandemic, for example, by taking over some pandemic-related services:

*‘Actually the different specialist groups should have been involved more, even if it is not their specialist area.* […] *If there is a pandemic and you realise that certain special examinations no longer need to be performed.* […] *That the healthcare system itself then says: “We activate this human resource, we will contact them and ask if they can join the normal pandemic care”.’* (GP 7)

Others wanted the health authorities to take on more pandemic-related tasks themselves, such as swabbing, so that GPs and MPAs could concentrate on general health care in the future:

*‘I called up the* [local] *health authority and said: “You should open an extra office downstairs with two nurses and do PCR swabs.”* […] *That would have been a much better system* […] *because patients would have continued to receive their normal general primary care here and we would not actually have spent half the day doing the work of the health authority.’* (MPA 11)

As practice owners, GPs had to weigh the medical services they offered against the financial realities of their practice. As a result, some were compelled to focus more narrowly on what was strictly medically necessary than before:

*‘However, we have of course tried to carry out more evidence-based and medically sensible activities, regardless of the financial costs or lower income we generate.’* (GP 5)

One example of this consideration was the care of chronically ill patients. In Germany, every patient must be seen by a doctor, even for the provision of a repeat prescription, to trigger case-related payment at the usual interval of every 3 months. These rules were temporarily relaxed during the pandemic. GP practices decided to evaluate the need for follow-up appointments individually and discussed it with patients, and they wanted to maintain this after the pandemic:

*‘We have actually realised that some of the intervals we have planned for reappointments with patients can be longer for some patients* […] *Formerly, we have appointed a lot of people on a quarterly basis, and now we feel it is quite possible to do it every 6 months.’* (GP 7)

## Discussion

### Summary

The results of this study highlight the significant challenges faced by GPs and MPAs during the pandemic, particularly in terms of their roles, collaboration with other healthcare professionals, and navigating financial and administrative pressures. The interviewees felt an increased sense of responsibility, with some finding greater value in their roles and others feeling exhausted. MPAs felt unseen by political decision makers and society compared to nurses in hospitals.

The interviewees expressed frustration with taking on the tasks of other healthcare professionals and institutions, and with limited interaction with them. They called for better communication in general and more collaboration, particularly in managing pandemic-related tasks.

Various in-practice procedures that were adapted due to the pandemic and will be retained after the pandemic were described. The switch to telemedicine and the corresponding remuneration was helpful. They reflected on what should have been done differently by others or within the healthcare system: the added administrative burden and unclear responsibilities (for example, related to COVID-19 vaccinations) created significant stress. One key concern was the lack of timely communication from authorities, which created difficulties in adapting to changing regulations. GPs had to balance patient care with the financial viability of their practices, making decisions about the necessity of services such as the frequency of follow-up appointments.

### Strengths and limitations

We included interviewees from two professions essential to the provision of care in general practices, thus giving a voice to both physicians and non-physician healthcare staff. By recruiting participants from rural and urban regions in three different federal states in Germany, different care settings were included, and the perspectives of healthcare professionals from these regions were considered.

Our analysis adds to the existing literature an overall impression of the pandemic period and the first period after the official end of the pandemic. At the time of the interviews, the GPs and MPAs had between 2.5 and 3.5 years of experience working during the pandemic. Looking back, they were now able to conclude how best to maintain GP care, what they wished for as appreciation and support from political decision makers, and for collaboration with other healthcare professionals and institutions.

There are few limitations to our study. We did not address GPs’ and MPAs’ experiences of any specific pandemic waves but instead asked broad questions about the entire period up to the time of interviews or the end of the pandemic — the interviews were conducted during different phases of the pandemic.

Selection bias cannot be ruled out since recruitment was carried out via the RESPoNsE PBRN, which may have led to practices coming forward that were committed to and interested in research. We tried to minimise this by also selecting practices that were not part of the RESPoNsE PBRN.

Some aspects of our interviews are specific to Germany, so transferability to other countries may be limited for these points. These include billing modalities and specific bureaucratic procedures.

### Comparison with existing literature

Our study is distinguished by the perspective of GPs and MPAs whose assessments based on their respective experiences were very similar: GPs showed high personal commitment during the pandemic and understood their key role in the pandemic response.^22^ The high workloads of general practice teams have been reported in various publications. Practices have reported that the amount of work was too much to manage^9,15^ and impacted GPs' wellbeing^23^ due to patient flow, bureaucracy, and the resulting extra hours.^7^ Our results highlight that through the course of the pandemic, some maintained a relatively positive outlook on their role, while others felt the need to set boundaries for their health.

A few studies focus on the perspectives of MPAs, nurses in general practices, or medical office assistants.^13,24–26^ MPAs reported high workload at the beginning of the pandemic,^24^ and the effects of overload are still noticeable after the pandemic.^13^ The responsibilities intensified^25^ and tasks were shifted towards them.^27^ While other research has reported that MPAs often expressed a lack of recognition from GPs,^28^ our study showed that MPAs felt mostly overlooked by policymakers and wider society, especially compared to nurses in hospitals. This may be specific to Germany, but it sheds light on the needed recognition of this important health professional group.

A lack of coordination and support from other healthcare providers has been described previously, including delays in support from health authorities^29^ and disrupted coordination with hospitals and secondary care specialists,^27^ while other research has highlighted an intensified collaboration between GPs, out-of-hours services, and other healthcare professionals.^5^ The interviewees emphasised that their high workload was partly due to additional tasks taken over from other health professionals and institutions. The intensity of their disappointment towards their colleagues has not yet been reported in the literature.

The shift of patient contacts to telephone^28^ or video consultations^5^ has been described elsewhere. Some also mentioned the need for funding models rewarding the providers for telehealth,^30^ while our interviewees described the remuneration of teleconsultation as key to expend the offer. Whereas previous research has reported general satisfaction with remote consultations during the pandemic,^28^ our participants emphasised their potential to facilitate work in general practice under pandemic conditions.

General practice teams felt that official information lacked consistency, and instructions were often unclear and differed between institutions. Interviewees from both professional groups underlined the need to inform practices at an early stage so adjustments could be made before the public demanded them. Although there are some positive examples of top-down and bottom-up information cascades,^31^ there still is a lack of clear responsibilities, fast communication channels, and easily accessible information.^22,32^

While a lack of or confusing information flows and unclear responsibilities are mentioned as stressors in other publications,^33,34^ the burden of additional administrative work and documentation requirements during the pandemic is rarely mentioned. This could be a specific aspect of the German healthcare system. Additionally, the idea of involving specialists in pandemic-related tasks has, to our knowledge, not been mentioned so far, with the focus instead being on nursing staff that could help with care delivery.^34^

Some systemic measures are currently being implemented in Germany that are intended to help improve pandemic preparedness, either directly or indirectly: the revision of the National Pandemic Plan in 2024^35^ and the Ministry of Health’s digitalisation strategy, with the introduction of electronic patient records and the initiation of digital communication in the healthcare system.^36^ The current government is also discussing the establishment of a commission to review the COVID-19 pandemic.^37^

### Implications for research and practice

Future research should explore ways to enhance interdisciplinary and interprofessional collaboration, and improve communication and information flow between GP practices, specialist practices, hospitals, and health authorities, particularly in emergency situations, to assure better pandemic preparedness in the future. The early involvement of GPs in decision making and better support for MPAs from health authorities, particularly during crises, should be evaluated.

Our findings highlight the need to ensure healthcare provision during pandemics, for example, by covering the costs of telemedicine. They also point to the importance of optimising task distribution within the healthcare system during crises, such as shifting certain pandemic-related responsibilities to specialists and health authorities to maintain general patient care. Furthermore, policymakers should be urged to support GP practices with clear responsibilities and structured information flows, particularly in times of crisis. It remains uncertain to what extent lessons from the pandemic will be integrated into future strategies to improve pandemic preparedness.

## Data

An overview of the themes with definitions and illustrative quotes is available from the corresponding author on reasonable request.
